# Efficient genome editing with CRISPR/Cas9 in *Pleurotus ostreatus*

**DOI:** 10.1186/s13568-021-01193-w

**Published:** 2021-02-20

**Authors:** Tatpong Boontawon, Takehito Nakazawa, Chikako Inoue, Keishi Osakabe, Moriyuki Kawauchi, Masahiro Sakamoto, Yoichi Honda

**Affiliations:** 1 Graduate School of Agriculture, Kyoto University, Oiwakecho, Kitashirakawa, Sakyo-ku, 606-8502 Kyoto, Japan; 2 Graduate School of Technology, Industrial and Social Sciences, Tokushima University, 770-8503 Tokushima, Japan

**Keywords:** Agaricomycete, Mushroom, *fcy1*, *pyrG*, Genome editing

## Abstract

*Pleurotus ostreatus* is one of the most commercially produced edible mushrooms worldwide. Improved cultivated strains with more useful traits have been obtained using classical breeding, which is laborious and time-consuming. Here, we attempted efficient gene mutagenesis using plasmid-based CRISPR/Cas9 as the first step for non-genetically modified (non-GM) *P. ostreatus* generation. Plasmids harboring expression cassettes of Cas9 and different single guide RNAs targeting *fcy1* and *pyrG* were individually transferred into fungal protoplasts of the PC9 strain, which generated some strains exhibiting resistance to 5-fluorocytosine and 5-fluoroorotic acid, respectively. Genomic PCR followed by sequencing revealed small insertions/deletions or insertion of a fragment from the plasmid at the target site in some of the drug-resistant strains. The results demonstrated efficient CRISPR/Cas9-assisted genome editing in *P. ostreatus*, which could contribute to the molecular breeding of non-GM cultivated strains in the future. Furthermore, a mutation in *fcy1* via homology-directed repair using this CRISPR/Cas9 system was also efficiently introduced, which could be applied not only for precise gene disruption, but also for insertions leading to heterologous gene expression in this fungus.

**Supplementary Information:**

The online version contains supplementary material available at 10.1186/s13568-021-01193-w.

## Introduction

One-fifth of the discovered fungal species are grouped in the *Agaricomycetes* clade from *Basidiomycota * (Kirk et al. 2008). Some of these fungi form relatively large multicellular structures such as fruiting bodies or mushrooms for their sexual reproduction, which in some cases have been utilized as human foods for many years. Examples of famous edible mushrooms in the *Agaricomycetes* are *Pleurotus ostreatus* (oyster mushroom), *Agaricus bisporus* (champignon), and *Boletus edulis* (porcini). Furthermore, in terms of medicinal properties, *A. bisporus* has been suggested to cure microbial diseases and cancer (Bhushan and Kulshreshtha 2018). An aqueous extract of *Lentinula edodes* (shiitake) could inhibit human breast cancer and stimulate the immune system (Israilides 2008). Thus, fungi from the *Agaricomycetes* class are worth studying because of their many advantages to humans.

To generate highly valuable strains of cultivated mushrooms, breeding has been conducted (Sonnenberg et al. 2008; Chakravarty 2011). The typical breeding of cultivated mushrooms uses strain crossing to modify the characteristics of the organisms; useful traits from a specific donor are introduced into an acceptor to yield a new hybrid strain with better properties. However, to restore the quality of a commercial strain, repeated back-crossing is required (Sonnenberg et al. 2008), which is laborious and takes a long time. Moreover, most of the cultivated mushrooms have a tetrapolar mating system ruled by two unlinked mating loci *A* and *B* (Raudaskoski and Kothe 2010; Kües et al. 2011), which complicates crossing and breeding. Therefore, new methodologies for more efficient and simpler breeding are needed.

Molecular genetics may resolve the problems associated with classical breeding. Indeed, molecular marker-assisted selection was developed for mushroom breeding (Okuda et al.  2009; Dai et al. 2017). Furthermore, an efficient gene targeting method for gene disruption or modification using homologous recombination is available in *P. ostreatus* (Salame et al. 2012), one of the most economically important cultivated mushrooms (Gregori 2007; Corrêa et al. 2016), and a white-rot fungus frequently used for molecular genetic studies on lignin degradation (Salame et al. 2013; Nakazawa et al. 2017; Yoav et al. 2018). Using this system, molecular breeding could be conducted to generate strains with desired phenotype(s) much more quickly and efficiently. Recently, the generation of sporeless strains by disrupting *mer3* or *msh4* with this system was shown (Yamasaki et al. 2021). However, such strains are considered genetically modified organisms (GMOs) because of the introduction of non-endogenous DNA sequence(s). In many European and Asian countries, GM crops are fully or partially banned. For example, product mixtures with specific percentages of GMO crops are acceptable in some countries such as the Czech Republic and Spain (Mahaffey et al. 2016), while all the GMO crops are prohibited in Germany and France. Therefore, to generate cultivated strains that are more easily accepted by the societies, new tools for the molecular breeding of non-GM mushrooms need to be developed.

The clustered regularly interspaced short palindromic repeat (CRISPR)/CRISPR-associated protein 9 (Cas9), which is an adaptive immune system found in archaea and bacteria (Ishino et al. 1987; Jinek et al. 2012; Song et al. 2019), has been recently utilized as a versatile gene-targeting tool. The Cas9 endonuclease is guided to a targeted chromosome site by a 20-bp single guide RNA (sgRNA), which results in the cleavage of genomic DNA at a specific site on the chromosome, followed by non-homologous end joining (NHEJ)-mediated repair. This sometimes introduces mutations at the target site due to errors in the repair process. Introducing the Cas9-sgRNA ribonucleoprotein (RNP) complex into mushroom strains would allow targeted gene mutagenesis without the introduction of non-endogenous DNA sequence for efficient molecular breeding. In 2016, it was reported that the well-known white button mushroom (*Agaricus bisporus*) with a CRISPR/Cas9 edited genome escaped GMO regulation of USDA in a news article of Nature (Waltz 2016). Thus, this system can be used to generate new-type of non-GM mushroom strains that can be more readily accepted. In this study, we demonstrate efficient CRISPR/Cas9-assisted gene mutagenesis in *P. ostreatus* by introducing a plasmid with Cas9 and sgRNA, which is a first step toward molecular breeding of non-GM mushrooms using CRISPR/Cas9.

## Materials and methods

### Strains, culture conditions, and genetic techniques

*Pleurotus ostreatus* monokaryon strain PC9 (Spanish Type Culture Collection accession number CECT20311), and strains used in this study are listed in Table 1. Yeast and malt extract with glucose (YMG) medium (Rao and Niederpruem 1969) solidified with 2 % (w/v) agar was used for routine cultures. The cultures were maintained at 28°C under continuous darkness, unless otherwise stated. To grow uracil and uridine auxotrophic strains, 0.18 mM uracil and 20 mM uridine were added to YMG medium (YMGUU), if necessary. Either 0.1 % (w/v) 5-FC or 5-FOA was added to the medium when necessary.

**Table 1:** *P. ostreatus* strains used in this study

Strain	Genotype/description	Source
PC9	*A2B1*/5-FC and 5-FOA-sensitive (Nakazawa et al. 2016)	CECT20311; Larraya et al. (1999)
20b	*A2B1 ku80*::*Cbx*^*R*^/5-FC- and 5-FOA-sensitive	Salame et al. (2012)
fc0-3	*A2B1 fcy1-1*/a 5-FC-resistant strain obtained after introducing pCcPef3-126-*fcy1*sg1	This study
fc1-3	*A2B1 fcy1-2*/a 5-FC-resistant strain obtained after introducing pCcPef3-126-*fcy1*sg2	This study
fc1-4	*A2B1 fcy1-3*/a 5-FC-resistant strain obtained after introducing pCcPef3-126-*fcy1*sg2	This study
fc1-5	*A2B1 fcy1-4*/a 5-FC-resistant strain obtained after introducing pCcPef3-126-*fcy1*sg2	This study
fc2-1	*A2B1 fcy1-5*/a 5-FC-resistant strain obtained after introducing pCcPef3-126-*fcy1*sg2	This study
fc2-2	*A2B1 fcy1-6*/a 5-FC-resistant strain obtained after introducing pCcPef3-126-*fcy1*sg2	This study
py1-2	*A2B1* /a 5-FOA-resistant strain obtained after introducing pCcPef3-126-*pyrG*sg1	This study
py1-4	*A2B1 pyrG-3*/a 5-FOA-resistant strain obtained after introducing pCcPef3-126-*pyrG*sg1	This study
py1-6	*A2B1 pyrG-4*/a 5-FOA-resistant strain obtained after introducing pCcPef3-126-*pyrG*sg1	This study
py1-9	*A2B1* /a 5-FOA-resistant strain obtained after introducing pCcPef3-126-*pyrG*sg1	This study
py1-10	*A2B1 pyrG-5*/a 5-FOA-resistant strain obtained after introducing pCcPef3-126*-pyrG*sg1	This study
py1-14	*A2B1 pyrG-6*/a 5-FOA-resistant strain obtained after introducing pCcPef3-126*-pyrG*sg1	This study
py1-17	*A2B1 pyrG-7*/a 5-FOA-resistant strain obtained after introducing pCcPef3-126-*pyrG*sg1	This study
hr1	*A2B1 ku80*::*Cbx*^*R*^ *fcy1-7*/a 5-FC-resistant strain obtained after introducing pCcPef3-126-*fcy1*sg2 and donor DNA with homology arms of 0.5 kb	This study
hr2	*A2B1 ku80*::*Cbx*^*R*^ *fcy1-7*/a 5-FC-resistant strain obtained after introducing pCcPef3-126-*fcy1*sg2 and donor DNA with homology arms of 0.5 kb	This study
hr3	*A2B1 ku80*::*Cbx*^*R*^ *fcy1-7*/a 5-FC-resistant strain obtained after introducing pCcPef3-126-*fcy1*sg2 and donor DNA with homology arms of 0.5 kb	This study
hr4	*A2B1 ku80*::*Cbx*^*R*^ *fcy1-7*/a 5-FC-resistant strain obtained after introducing pCcPef3-126-*fcy1*sg2 and donor DNA with homology arms of 0.5 kb	This study
hr5	*A2B1 ku80*::*Cbx*^*R*^ *fcy1-7*/a 5-FC-resistant strain obtained after introducing pCcPef3-126-*fcy1*sg2 and donor DNA with homology arms of 1 kb	This study
hr6	*A2B1 ku80*::*Cbx*^*R*^ *fcy1-7*/a 5-FC-resistant strain obtained after introducing pCcPef3-126-*fcy1*sg2 and donor DNA with homology arms of 1 kb	This study
hr7	*A2B1 ku80*::*Cbx*^*R*^/a 5-FC-resistant strain obtained after introducing pCcPef3-126-*fcy1*sg2 and donor DNA with homology arms of 0.2 kb	This study
hr8	*A2B1 ku80*::*Cbx*^*R*^/a 5-FC-resistant strain obtained after introducing pCcPef3-126-*fcy1*sg2 and donor DNA with homology arms of 0.5 kb	This study
hr9	*A2B1 ku80*::*Cbx*^*R*^ *fcy1-7*/a 5-FC-resistant strain obtained after introducing pCcPef3-126-*fcy1*sg2 and donor DNA with homology arms of 0.5 kb	This study
hr10	*A2B1 ku80*::*Cbx*^*R*^ *fcy1-7*/a 5-FC-resistant strain obtained after introducing pCcPef3-126-*fcy1*sg2 and donor DNA with homology arms of 0.5 kb	This study
hr11	*A2B1 ku80*::*Cbx*^*R*^ *fcy1-7*/a 5-FC-resistant strain obtained after introducing pCcPef3-126-*fcy1*sg2 and donor DNA with homology arms of 1 kb	This study
hr12	*A2B1 ku80*::*Cbx*^*R*^/a 5-FC-resistant strain obtained after introducing pCcPef3-126-*fcy1*sg2 and donor DNA with homology arms of 0.2 kb	This study
hr13	*A2B1 ku80*::*Cbx*^*R*^ *fcy1-7*/a 5-FC-resistant strain obtained after introducing pCcPef3-126-*fcy1*sg2 and donor DNA with homology arms of 0.5 kb	This study
hr14	*A2B1 ku80*::*Cbx*^*R*^ *fcy1-7*/a 5-FC-resistant strain obtained after introducing pCcPef3-126-*fcy1*sg2 and donor DNA with homology arms of 0.5 kb	This study
hr15	*A2B1 ku80*::*Cbx*^*R*^ *fcy1-7*/a 5-FC-resistant strain obtained after introducing pCcPef3-126-*fcy1*sg2 and donor DNA with homology arms of 0.5 kb	This study
hr16	*A2B1 ku80*::*Cbx*^*R*^ *fcy1-7*/a 5-FC-resistant strain obtained after introducing pCcPef3-126-*fcy1*sg2 and donor DNA with homology arms of 1 kb	This study
hr17	*A2B1 ku80*::*Cbx*^*R*^ *fcy1-7*/a 5-FC-resistant strain obtained after introducing pCcPef3-126-*fcy1*sg2 and donor DNA with homology arms of 1 kb	This study

The transformation of *P. ostreatus* strains using the hygromycin resistance gene (*hph*) was performed using protoplasts prepared from mycelial cells as described by Salame et al. (2012).

### Design of sgRNAs targeting ***fcy1*or*pyrG***

The different sgRNA sequences used to target *fcy1* and *pyrG* (Nakazawa et al. 2016) [Protein ID: 89,004 and 83,414, respectively, in the genome database of *P. ostreatus* PC9 (https://mycocosm.jgi.doe.gov/PleosPC9_1/PleosPC9_1.home.html)] were designed based on on-target (Doench et al. 2014) and off-target (Xiao et al. 2014) scores calculated by the Focas UI website (http://focas.ayanel.com). The four sgRNA sequences were: *fcy1*sg1 (*fcy1*, nucleotide positions 32–51) and *fcy1*sg2 (*fcy1*, 92–111); *pyrG*sg1 (*pyrG*, 521–540) and *pyrG*sg2 (*pyrG*, 559–578), from the start codon (Additional file 1: Table S1).

### Plasmid construction

In this study, the vector pCcPef3-126 was used for genome editing (Additional file 1: Fig. S1) (Sugano et al. 2017; Nguyen et al. 2020). The basdiomycete codon optimized *Streptococcus pyogenes* Cas9 for with 3 × nuclear localization sequences (NLSs) and the sgRNA scaffold were driven by the *C. cinerea ef3* (an elongation factor 3) promoter and the *u6* promoter, respectively. The *hph* cassette from pPHT1 (Cumming et al. 1999) was used to confer resistance to hygromycin B on *P. ostreatus*. The double-stranded DNA, which was prepared by annealing two DNA oligonucleotides [primers CI19/CI20 (*fcy1*sg1) and CI21/CI22 (*fcy1*sg2) for *fcy1*; TB41/TB42 (*pyrG*sg1) and TB43/TB44 (*pyrG*sg2) for *pyrG* (Additional file 1: Table S1)], containing each sgRNA sequence was inserted into the *Bsa*I site of the linearized pCcPef3-126 vector using Golden Gate assembly (Engler 2008, 2009). The sgRNA insertion into plasmids was verified with Sanger sequencing. The resulting plasmids were named pCcPef3-126-*fcy1*sg1, pCcPef3-126-*fcy1*sg2, pCcPef3-126-*pyrG*sg1, and pCcPef3-126-*pyrG*sg2, respectively.

### Design of the donor DNA templates for homologous recombination

**Fig. 1:**
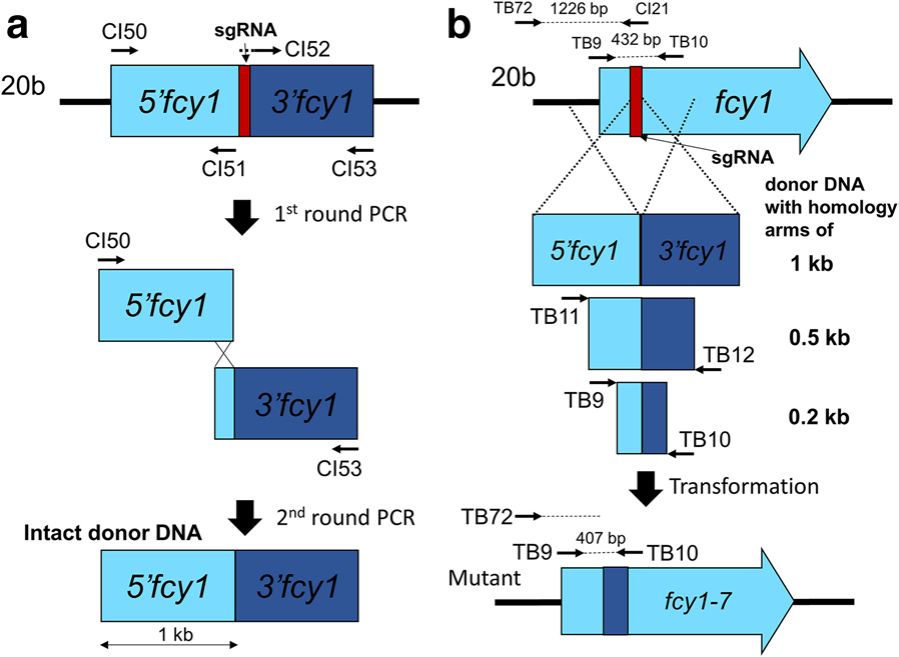
Targeted gene replacement using homologous recombination along with CRISPR/Cas9. **a** A schematic diagram of the procedure used to construct the donor DNA template that lacks the sgRNA sequence for homologous recombination. **b** A schematic diagram showing the method for *fcy1* mutation using CRISPR/Cas9 and donor DNA. PCR fragments with around 1 kb, 0.5 kb, and 0.2 kb each of right and left homologous arms were used as donor DNA temples. Black arrows display the primer pairs used for the PCR experiments in Additional file 1: Fig. S2C

For precise gene replacement using homologous recombination in the *fcy1* gene, donor DNA templates (with a 25-bp deletion of the *fcy1*sg2 sequence) with homology arms of 1 kb, 0.5 kb, or 0.2 kb, were constructed using overlap extension PCR. Approximately 1-kb genomic fragments containing 5’-upstream and 3’-downstream sequences were amplified using CI50/CI51 and CI52/CI53, respectively (Fig. 1a). These fragments were fused by CI50/CI53 to obtain the intact donor DNA, followed by amplification with TB11/TB12 and TB9/TB10 to reduce the length of homologous arms to 0.5 kb and 0.2 kb, respectively (Fig. 1b). The resulting fragments were designated as donor DNA with homology arms of 1 kb (2071 bp in total), 0.5 kb (1008 bp in total), and 0.2 kb (407 bp in total), respectively (Additional file 1: Table S2).

### Rapid colony PCR

To verify gene mutations, rapid colony PCR was performed. Genomic DNA was extracted with the thermolysis method described by Zhang et al. (2010) with some modifications. Briefly, a small agar plug (approximately 3 × 3 mm) covered with mycelium was placed into 200 µl of sterilized distilled water for removal of PCR inhibitors and briefly centrifuged at 13,200 ×*g* for 1 min. Then, 200 µl of DNA extraction buffer [50 mM sodium phosphate (pH 7.4), 1 mM EDTA, and 5 % (v/v) glycerol] was added, the mixture was incubated at 85 °C for 30 min, and then kept at − 20 °C until further use. In this study, we used the EmeraldAmp MAX PCR Master Mix (Takara Bio, Shiga, Japan) and KAPA2G Robust HotStart PCR kit (Nippon Genetics, Tokyo, Japan) for the PCR experiments.

### Genomic PCR to identify mutations

To examine the type of mutations, long-range PCR was performed. The high-quality fungal genomic DNA was extracted with the CTAB method (Zolan and Pukkila 1986; Muraguchi et al. 2003). Briefly, *P. ostreatus* strains were grown on YMG agar medium for 7 days and the mycelial cells were freeze-dried overnight using a small freeze-dryer (FDS-1000, EYELA, Tokyo, Japan). The disrupted lyophilized cells were resuspended in 900 µl of CTAB buffer [0.003 % (w/v) CTAB, 0.68 M NaCl, 50 mM Tris-HCl pH 8.0, and 10 mM EDTA] with 18 µl of 2-mercaptoethanol and incubated at 50 °C for 30 min. Next, 900 µl of chloroform was added, mixed by inversion, and then centrifuged at 13,200 × *g* for 5 min. The supernatant was mixed with chloroform and phenol (2:1:1), and centrifuged. Then, 600 µl of isopropanol and 30 µl of 5 M NaCl were added to the supernatant. The mixture was centrifuged at 13,200 × *g* for 15 min, and the DNA pellet was washed once with 1 ml of cold 70 % ethanol, air dried, and resuspended in 50 µl of TE buffer (10 mM Tris-HCl pH 8.0 and 1 mM EDTA) at a final concentration of 10 µg/ml of RNase A. In this study, we used the Proofreading polymerase KOD FX Neo (Toyobo, Osaka, Japan) with a step-down cycle, as indicated by the manufacturer for long-distance PCR amplification. Then, the PCR fragments were purified using the FastGene Gel/PCR Extraction Kit (Nippon Genetics, Tokyo, Japan), followed by Sanger sequencing.

## Results

### **Expressing Cas9 and sgRNA by plasmid introduction frequently confers resistance to 5-FC and 5-FOA on the*P. ostreatus*PC9 strain**

To examine the efficiency of CRISPR/Cas9-assisted gene mutagenesis by introducing a plasmid expressing Cas9 and sgRNA, the *fcy1* and *pyrG* genes were selected as targets to be mutated because their single-gene mutations confer resistance to 5-FC and 5-FOA in *P. ostreatus*, respectively (Nakazawa et al. 2016). This allowed us to identify the mutants easily and efficiently by examining resistance/sensitivity. The plasmids pCcPef3-126-*fcy1*sg1, pCcPef3-126-*fcy1*sg2, pCcPef3-126-*pyrG*sg1, and pCcPef3-126-*pyrG*sg2 were separately introduced into the *P. ostreatus* PC9 host strain (Table 1) to obtain hygromycin-resistant transformants. These plasmids express *fcy1*- or *pyrG-*targeting sgRNAs (*fcy1*sg1, *fcy1*sg2, *pyrG1*sg1, and *pyrG*sg2), respectively, together with Cas9 and a hygromycin phosphotransferase (Hph) that confers resistance to hygromycin B. The empty vector pCcPef3-126, which expresses an sgRNA scaffold without the targeting sequence, was also introduced as a control. A total of 14 hygromycin-resistant transformants were obtained in three independent experiments by introducing the empty vector, all of which did not grow on YMG with 0.1 % (w/v) of 5-FC or 5-FOA (Table 2). This result showed that introducing the empty vector pCcPef3-126 rarely confers resistance to 5-FC and 5-FOA on *P. ostreatus* PC9. On the other hand, one out of five (20 %) and 22 out of 28 (78.6 %) hygromycin-resistant transformants, obtained by introducing pCcPef3-126-*fcy1*sg1 and pCcPef3-126-*fcy1*sg2, respectively, exhibited resistance to 5-FC. Eight out of 17 (47.1 %) and two out of seven (28.6 %) hygromycin-resistant transformants, obtained by introducing pCcPef3-126-*pyrG*sg1 and pCcPef3-126-*pyrG*sg2, respectively, exhibited resistance to 5-FOA. Similar results were obtained in the replicate experiments (Table 2). These results suggested that 5-FC- or 5-FOA-resistant *P. ostreatus* strains can be frequently generated by introducing plasmids using the CRISPR/Cas9 system; the frequency/efficiency seemed to depend on the target sequence in sgRNA.

**Table 2:** Comparison of transformants and mutation frequency obtained using different sgRNAs targeting *fcy1* or *pyrG*

Plasmid with sgRNA	Hygromycin-resistant strains			5-FC- or 5-FOA-resistant strains			*fcy1* or *pyrG* mutants by PCR^a^		
	Rep 1	Rep 2	Rep 3	Rep 1	Rep 2	Rep 3	Rep 1	Rep 2	Rep 3
Empty vector	2	4	8	0^b^	0^b^	0^b^	–	–	–
*fcy1* sgRNA No. 1	5	11	18	1 (20%)^c^	0	0	1/1	–	–
*fcy1* sgRNA No. 2	28	23	34	22 (78.6%)^c^	15 (65.2%)^c^	30 (88.2%)^c^	5/5	4/5	4/5
*pyrG* sgRNA No. 1	17	19	–	8 (47.1%)^c^	18 (94.7%)^c^	–	7/8	10/10	–
*pyrG* sgRNA No. 2	7	7	–	2 (28.6%)^c^	2 (28.6%)^c^	–	2/2	2/2	–

^a^The number of mutants which can be identified by difference size, or no amplified, bands compared to wild-type control were observed in the genomic PCR experiment (Additional file 1: Fig. S2A and B)

^b^The number of 5-FC- or 5-FOA-resistant strains

^c^Percentage of 5-FC- or 5-FOA-resistant strains from the total number of hygromycin-resistant strains

### **Identification of small deletion mutations in*fcy1*and*pyrG***.

Next, we verified that the obtained 5-FC- and 5-FOA-resistant strains were *fcy1* and *pyrG* mutants, respectively. An NHEJ-mediated gene mutation using the CRISPR/Cas9 system typically produces small insertions or deletions (indels), such as the insertion of several nucleotides and deletion from a single nucleotide to several hundreds of nucleotides at/around a target site. Therefore, we first attempted to PCR-amplify genomic fragments of the partial open reading frames (ORFs) of *fcy1* (432 bp) and *pyrG* (356 bp) containing the target sequences of sgRNA using primer pairs TB9/TB10 and TB53/TB54, respectively. The primer pair FY15/FY16, which amplifies a 430-bp genomic fragment from the ORF of *mer3* encoding an ATP-dependent DNA helicase (Protein ID 82,484 in the JGI genome database) was also used as a positive control.

**Fig. 2:**
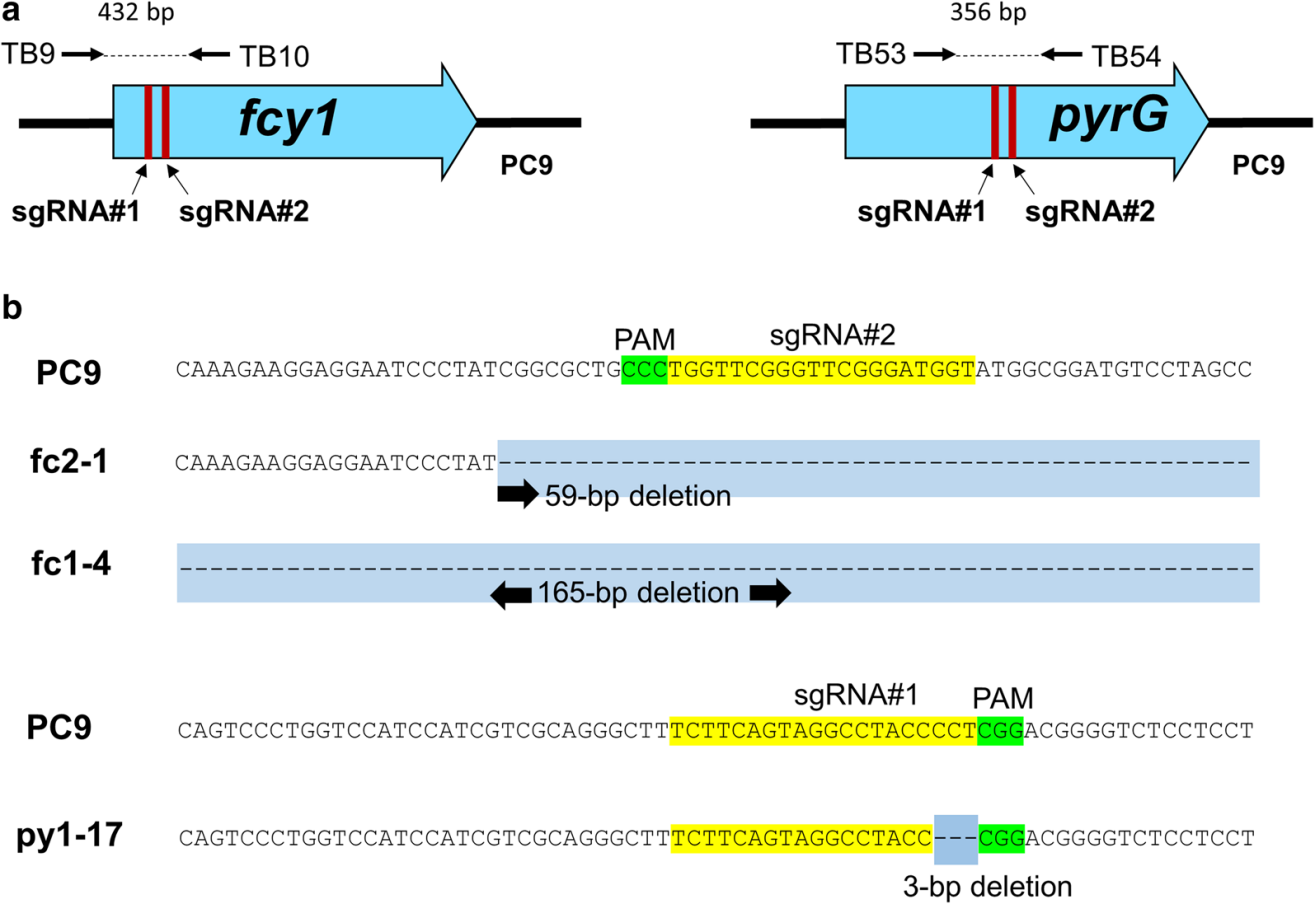
Identification of *fcy1* and *pyrG* mutations in some strains. **a** A schematic diagram of the *fcy1* and *pyrG* loci in the PC9 host strain with sgRNA recognition sites. The dash lines indicate the region amplified by genomic PCR. Black arrows display the primer pairs used for the PCR experiments in Additional file 1: Fig. S2A and B. **b** DNA sequencing to identify small indel mutations in *fcy1* or *pyrG* mutants. For highlights in the nucleotide sequence: yellow shades indicate sgRNA, green shades indicate a protospacer adjacent motif (PAM) sequence, and dash lines indicate deletion

Some 5-FC- or 5-FOA-resistant strains were randomly selected for examination of gene mutation using genomic PCR (Table 2), and the result of agarose gel electrophoresis in ten strains each of 5-FC and 5-FOA resistance was shown in the supplementary figure (Additional file 1: Fig. S2A and B, respectively). The PCR product was amplified from the ten 5-FC-resistant strains and the ten 5-FOA-resistant strains used (lanes 1–10 in Fig. S2A and B) when the primer pair FY15/FY16 was used. As shown in Additional file 1: Fig. S2A and B, similar to the parental control strain PC9, the fragment was amplified from two out of ten 5-FC-resistant strains (lanes 4 and 6 in Additional file 1: Fig. S2A; strains fc1-4 and fc2-1, respectively) and one out of ten 5-FOA-resistant strains (lane 8 in Additional file 1: Fig. S2B; strain py1-17) when the primer pairs TB9/TB10 and TB53/TB54 were used, respectively. The sizes of the fragments amplified from fc1-4 and fc2-1 seemed to be different from that amplified from PC9 (Additional file 1: Fig. S2A), suggesting that small indels may have been introduced at the target sites of *fcy1*/*pyrG*, at least in fc1-4 and fc2-1. To confirm if small indels were introduced at the target sites in the three resistant strains from which the genomic fragment was amplified (fc1-4, fc2-1, and pyl-17), the nucleotide sequences of the PCR-amplified fragments were analyzed using TB9 or TB10 for *fcy1*, and TB53 or TB54 for *pyrG*. The results revealed 165-bp and 59-bp deletions around the target site of *fcy1*sg2 in fc1-4 and fc2-1, respectively. A 3-bp deletion mutation at the target site of *pyrG*sg1 in the 5-FOA resistant strain, py1-17 (lane 8), was also revealed (Fig. 2b; Table 1). The mutations newly identified in fc1-4, fc2-1, and py1-17 were designated as *fcy1-3*, *fcy1-5*, and *pyrG-7*, respectively (Table 1). These results indicated that they are *fcy1* or *pyrG* mutants harboring small indel mutations; however, the mutations were not identified in many other strains from which the genomic fragment was not amplified.

### Identification of the CRISPR/Cas9 plasmid insertion at the target sites

Considering the fact that the PCR fragment was not amplified from many 5-FC- or 5-FOA-resistant strains (lanes 1–3, 5, 7–10 in Additional file 1: Fig. S2A; lanes 1–7, 9–10 in Fig. S2B), the mutations could have been introduced into the chromosomes of these strains in different manners. We hypothesized that the introduced plasmids had been inserted at the target site of each sgRNA. Based on this assumption, eight pairs of primers were designed to hybridize to the genomic region, which is located approximately 200-bp apart from the sgRNA target site in the first primer (primer TB9 or TB10 for *fcy1*; TB53 or TB54 for *pyrG*), and the *C. cinerea β1-tubulin* promoter or terminator in the plasmids in the other primer (primer TN40 or TN46). The set of primers TB9/TN40, TB9/TN46, TB10/TN40, and TB10/TN46 for *fcy1*, and TB53/TN40, TB53/TN46, TB54/TN40, and TB54/TN46 for *pyrG*, were used for genomic PCR on six 5-FC- and six 5-FOA-resistant strains, respectively (Additional file 1: Table S2). In the case of *fcy1*, around 6 kb, 1.5 kb, 7 kb, and 10 kb fragments were amplified from four 5-FC-resistant strains (namely fc0-3, fc1-3, fc2-2, and fc1-5, respectively) when the primer pairs TB10/TN40, TB10/TN46, TB9/TN40, and TB9/TN46 were used, respectively. In the case of *pyrG*, around 1.7 kb, 2.5 kb, 2.5 kb, 5 kb, 8 kb, and 6 kb fragments were amplified from six 5-FOA-resistant strains (namely py1-2, py1-4, py1-6, py1-9, py1-10, and py1-14, respectively) when the primer pairs TB53/TN46, TB53/TN46, TB53/TN46, TB53/TN46, TB54/TN46, and TB53/TN46, were used, respectively. These PCR fragments were not amplified from their parental control strain, PC9, when these sets of primers were used (data not shown). These results suggested that the introduced plasmids had been inserted at the target sites of *fcy1*/*pyrG*, at least in the ten strains from which the fragment was amplified.

**Fig. 3:**
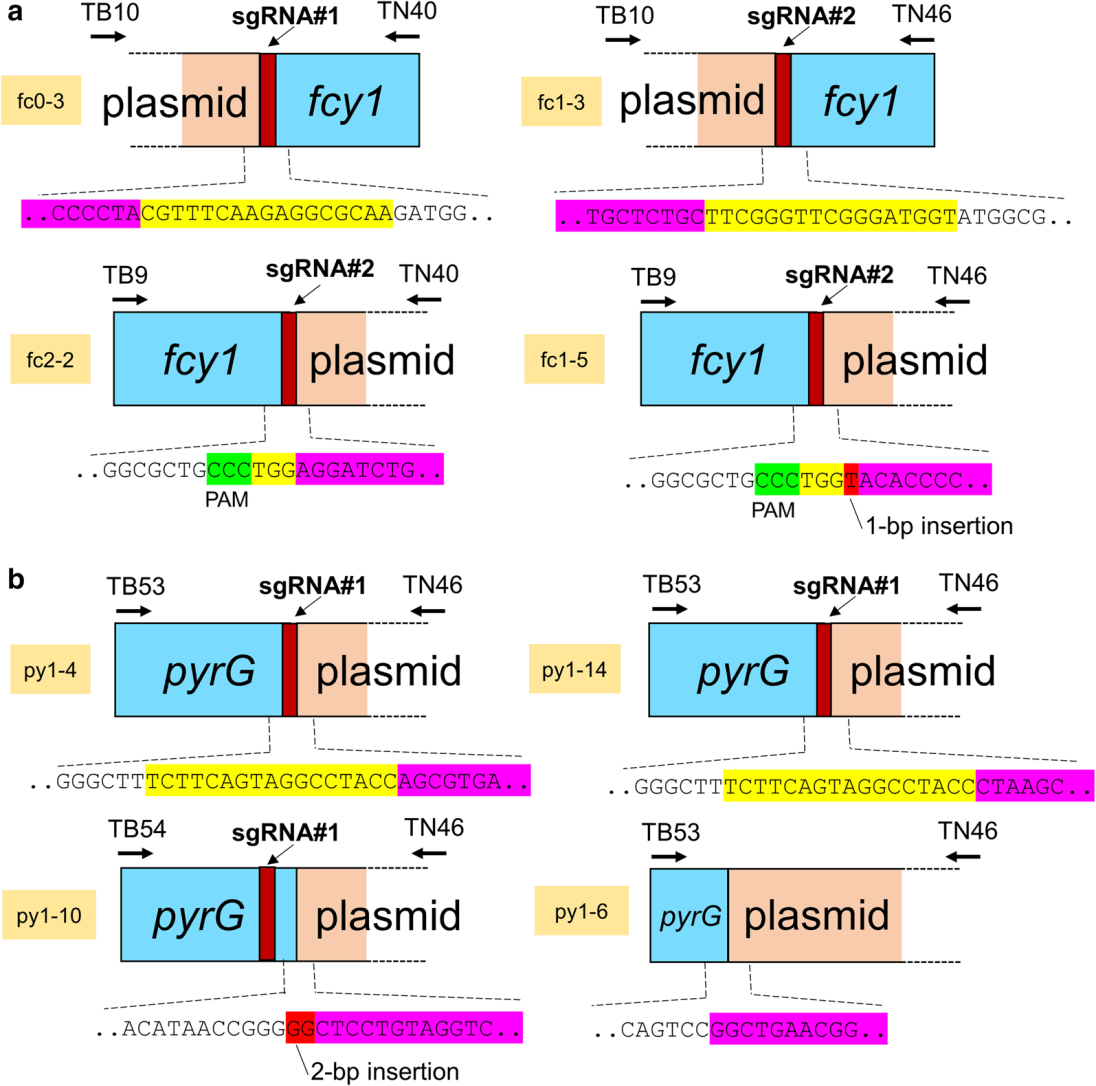
Identification of insertional mutations in some *fcy1* or *pyrG* mutants. Schematic diagrams showing how the introduced plasmids had been inserted at the target sites of *fcy1* (**a**) or *pyrG* (**b**). For highlights in the nucleotide sequence: yellow shades indicate sgRNA, green shades indicate PAM sequence, pink shades indicate plasmid sequence, and red shades indicate insertion

To confirm the possibility of the insertional mutation, the PCR-amplified fragments were subjected to DNA sequencing using primers TB9 or TB10 for *fcy1*, and TB53 or TB54 for *pyrG*. The results demonstrated the insertion of the introduced plasmids at the target sites of the three 5-FC-resistant strains (strain fc0-3 generated by introducing the pCcPef3-126-*fcy1*sg1, and strains fc1-3 and fc2-2 by pCcPef3-126-*fcy1*sg2), and the plasmid insertion altogether with a 1 bp insertion at the target site of *fcy1*sg2 in one 5-FC-resistant strain, fc1-5 (Fig. 3a). As for *pyrG* mutations in the 5-FOA-resistant strains, the mutations were successfully identified in the four mutants. The insertion of the introduced plasmid at the target site of *pyrG*sg1 in the three 5-FOA-resistant strains, namely py1-4, py1-6, and py1-14, and the plasmid insertion altogether with a 2 bp insertion at the target site of *pyrG*sg1 in the one 5-FOA-resistant strain, py1-10, are shown in Fig. 3b. We also attempted to identify the insertional mutations of the two 5-FOA-resistant strains, py1-2 and py1-9; however, the PCR-amplified fragments could not be sequenced using the TB53 primer. The mutations in these strains, namely fc0-3, fc1-3, fc1-5, fc2-2, py1-4, py1-6, py1-10, and py1-14, were designated as *fcy1-1*, *fcy1-2*, *fcy1-4, fcy1-6*, and *pyrG-3*–*6*, respectively (Table 1). These results suggested that the insertional mutation was introduced into *fcy1* and *pyrG* in some of the 5-FC- and 5-FOA-resistant strains, respectively.

### **Targeted gene replacement using homologous recombination with a donor DNA template**

The gene mutations identified in the above experiments were most likely introduced by NHEJ after the double-strand DNA break (DSB), which was caused by the expressed Cas9-sgRNA complexes. However, the NHEJ-mediated mutations are generally unpredictable, which precludes us from generating strains with the desired mutations using CRISPR/Cas9. For precise gene replacement and introduction of the targeted mutation, CRISPR/Cas9 with a donor DNA template may be used to induce homology-directed repair (HDR)-mediated mutations in *P. ostreatus*. First, to examine whether the ORF of *fcy1* can be frequently replaced with that of donor DNA (with the 25-bp deletion in the ORF containing the *fcy1*sg2 sequence; Fig. 1a) using CRISPR/Cas9-assisted homologous recombination, the plasmid pCcPef3-126-*fcy1*sg2 was introduced with or without a donor DNA template with homology arms of 1 kb (Fig. 1b). In this experiment, strain 20b (a *ku80* disruptant from PC9), but not PC9, was used because the *ku80* deficiency impairs the NHEJ pathway and decreases the frequency of NHEJ-mediated ectopic integration (Salame et al. 2012). Four hygromycin-resistant transformants were obtained from three independent experiments by introducing only the plasmid pCcPef3-126-*fcy1*sg2, all of which did not grow on YMG with 0.1 % (w/v) 5-FC (Table 3). This result indicated that introducing the plasmid pCcPef3-126-*fcy1*sg2 alone rarely confers resistance to 5-FC on the *P. ostreatus* 20b strain, a result inconsistent with that of the wild-type strain, as introducing pCcPef3-126-*fcy1*sg2 onto PC9 frequently conferred resistance to 5-FC (Table 2). This may be due to our hypothesis that the introduced plasmid was frequently inserted at the target site by NHEJ in PC9, but not in 20b due to its reduced NHEJ activity/pathway. Five out of six (83.3 %) hygromycin-resistant transformants, obtained by introducing pCcPef3-126-*fcy1*sg2 along with the donor DNA template, exhibited resistance to 5-FC (Table 3). This result suggested that targeted gene replacement may occur frequently when the CRISPR/Cas9 plasmid is introduced into *P. ostreatus* 20b strains along with a DNA repair template.

**Table 3:** Comparison of transformants and mutation frequency obtained using CRISPR/Cas9 and donor DNA templates

Plasmid with sgRNA	Donor DNA template	Hygromycin-resistant strains				5-FC-resistant strains				*fcy1* mutant by PCR^a^			
		Rep 1	Rep 2	Rep 3	Total	Rep 1	Rep 2	Rep 3	Total	Rep 1	Rep 2	Rep 3	Total
*fcy1* sgRNA No. 2	–	0	2	2	4	0	0	0	0	0	0	0	0
	0.2 kb	0	2	2	4	0	1	1	2 (50%)^b^	0	0	0	0
	0.5 kb	4	5	4	13	4	3	3	10 (76.9%)^b^	4	2	3	9 (69.2%)^c^
	1 kb	2	2	2	6	2	1	2	5 (83.3%)^b^	2	1	2	5 (83.3%)^c^

^a^The number of mutants which can be identified by difference size, or no amplified, bands compared to wild-type control were observed in the genomic PCR experiment (Additional file 1: Fig. S2C)

^b^Percentage of 5-FC-resistant strains from the total number of hygromycin-resistant strains

^c^Percentage of *fcy1* mutants from the total number of hygromycin-resistant strains

Second, to examine the effect of homology arm length on frequency/efficiency, the plasmid pCcPef3-126-*fcy1*sg2 with reduced size of DNA repair templates (homology arms of 0.5 kb and 0.2 kb) was introduced into the 20b strain. As shown in Table 3, ten out of 13 (76.9 %) and two out of four (50.0 %) hygromycin–resistant strains, obtained in three independent experiments by introducing pCcPef3-126-*fcy1*sg2 with homology arms of 0.5 kb and 0.2 kb, respectively, exhibited resistance to 5-FC. These results suggested that the frequency of 5-FC resistance may be higher when using a longer DNA repair template with the CRISPR/Cas9 system.

We then verified that the *fcy1* sequence was replaced with the introduced donor DNA, as expected. The 17 5-FC-resistant strains obtained by introducing the pCcPef3-126-*fcy1*sg2 plasmid along with the donor DNA template with 1-kb homology arms (five strains, hr5, hr6, hr11, hr16, hr17), 0.5-kb one (ten strains, hr1–4, hr8, hr9, hr10, hr13–15), or 0.2-kb one (two strains, hr7 and hr12), were used for this experiment (Table 1). First, we examined whether a genomic fragment (1226 bp) was amplified from strains hr1–17 when the primer pair TB72/CI21 was used (Fig. 1b). This fragment was anticipated to be amplified from 20b, but not from strains into which the HDR-mediated *fcy1* mutation had been introduced, due to loss of the hybridization sequence from primer CI21 after the HDR-mediated mutation. As shown in Additional file 1: Fig. S2C, the expected fragment was amplified from the 20b strain (lane wt), but not from the 5-FC-resistant strains (lanes 1–17). This result suggested that the HDR-mediated mutation could be introduced into the genomes of all 5-FC-resistant strains.

Furthermore, the other primer pair TB9/TB10 was used to confirm whether a 432-bp PCR product was amplified from the wild type strain, while a shorter size of genomic fragment (407 bp) was amplified from the prospect mutant strains due to loss of a region containing the sgRNA recognition site on the DNA repair template (Fig. 1b). As shown in Additional file 1: Fig. S2C, the expected fragment was amplified from the 20b strain (lane wt), whereas the shorter fragment was amplified from 14 out of 17 5-FC-resistant strains (lanes 1–6, 9–11, 13–17; strains hr1–6, hr9–11, hr13–17, respectively). The mutations in these 14 strains were designated as *fcy1-7* (Table 1). This result indicated that all five, and nine out of ten 5-FC-resistant strains were *fcy1* disrupted when using the repair templates with homology arms of 1 kb and 0.5 kb, respectively (Table 3). Moreover, these results suggested that precise/targeted gene replacement can be performed using CRISPR/Cas9 along with a donor DNA template with at least 0.5-kb homology arms in *P. ostreatus*.

## Discussion

Here, we demonstrate efficient CRISPR/Cas9-assisted gene mutagenesis by plasmid introduction in *P. ostreatus*. In the *Agaricomycetes* class, CRISPR/Cas9-assisted genome editing was previously reported in *C. cinerea* and *Ganoderma lucidum* by plasmid introduction; however, the efficiencies/frequencies were 10.5–32.0 % (Sugano et al. 2017; Wang et al. 2020), which were much lower than those in this study (20–94.9 %). Therefore, the CRISPR/Cas9 system in *P. ostreatus* used in this study may currently be the most efficient among agaricomycetes. This new, efficient tool developed in *P. ostreatus* could be applied to molecular breeding as well as to studies on fruiting development and lignin degradation.

In this study, the plasmid pCcPef3-126 designed for CRISPR/Cas9 in *C. cinerea* (Sugano et al. 2017) was also available in *P. ostreatus*. Considering that the introduction of this plasmid into *P. ostreatus* PC9 resulted in the efficient introduction of gene mutations, all the expression cassettes for Cas9, Hph, and sgRNA work well not only in *C. cinerea* but also in *P. ostreatus* (both in the order *Agaricales*). Furthermore, the *hph* cassette in this plasmid, which is derived from the pPHT1 designed for hygromycin resistance transformation in *C. cinerea* (Cummings 1999), was also shown to be available for that in *Ceriporiopsis subvermispora*, which belongs to the order *Polyporales* (unpublished). This suggests that CRISPR/Cas9-assisted genome editing using the pCcPef3-126 plasmid can be applied in many other agaricomycetes with high economical and medicinal value, as long as hygromycin resistance transformation is available/developed.

We successfully identified *fcy1* or *pyrG* mutations in some of the genome-edited strains; small indels in three mutants, and insertion of the introduced plasmid in eight mutants. Small indels have been identified in genome-edited mutants of various fungi, including *C. cinerea* and *G. lucidum* (Sugano et al.  2017; Qin et al.   2017; Wang et al.   2020). However, to the best of our knowledge, the insertion of a plasmid sequence at the target site of sgRNA, which may be mediated by the NHEJ pathway, has not been previously reported, except for the intended plasmid insertion system in *Fusarium oxysporum* (Wang and Coleman 2019). Generally, the frequency of homologous recombination in agaricomycetes is much lower than that in ascomycetes (Nakazawa et al. 2011; Salame et al. 2012), which may be due to higher NHEJ activity in agaricomycetes. This could be the reason behind the insertional mutation in *P. ostreatus*. If this hypothesis is correct, genome-edited strains with insertional mutations may be obtained when pCcPef3-126-based CRISPR/Cas9 is performed in other agaricomycetes.

However, not all the mutations in the mutants examined in this study could be identified in the genomic PCR experiments. Therefore, the mutations might also be introduced differently. For example, the genomic sequence around the target site could be largely deleted, which would also cause the loss of the chromosomal region where the primers anneal, precluding the PCR-amplification of the fragment. Moreover, translocation is also likely to occur. These possibilities should be examined in future studies using Southern blot analyses and long-read whole-genome resequencing.

Furthermore, we demonstrate CRISPR/Cas9-assisted gene replacement via HDR in *P. ostreatus*. Conventional gene targeting experiments using homologous recombination have been performed with *P. ostreatus* 20b strain and 1.5–2 kb homology arms (Salame et al. 2012; Nakazawa et al. 2016), while *fcy1* mutants were successfully obtained when a shorter donor DNA template with homology arms of 1 kb and 0.5 kb was used in this study. Thus, CRISPR/Cas9-assisted replacement via HDR enables targeted gene replacement using a shorter homology arm, which may be more useful than the conventional method. Although the possibility of ectopic integration of the donor DNA as well as the introduced plasmid with the expression cassettes for sgRNA and Cas9 into the host chromosome cannot be excluded, this method could be more useful for expression cassette(s) insertion and gene knock-in/knock-out in molecular genetics studies of *P. ostreatus*.

In conclusion, this is the first report demonstrating genome editing using the CRISPR/Cas9 system in an edible mushroom. Future studies will focus on developing a marker-free CRISPR system for molecular breeding of non-GM mushrooms. However, the efficiency/frequency of gene mutagenesis with Cas9/sgRNA ribonucleoprotein (RNP) complex seems to be very low in the agaricomycete *S. commune* (Jan Vonk et al. 2019), suggesting that some difficulties may have to be overcome to establish marker-free CRISPR/Cas9 in agaricomycetes.

## Supplementary Information

**Additional file 1: Fig. S1.** A plasmid map of the pCcPef3-126 plasmid shows the components of the construct. **Fig. S2.** Agarose gel electrophoresis. Genomic PCR experiments examining/verifying *fcy1* (A) or *pyrG* (B) mutation. (C) Genomic PCR experiments confirming gene replacement in the 5-FC-resistant strains obtained after introducing the pCcPef3-126-*fcy1*sg2 plasmid with the donor DNA templates. Lane WT, the parental strain PC9 (A and B) and 20b (C) as a positive control; Lanes 1–10 (A and B), 5-FC- and 5-FOA- resistant strains, respectively; Lanes 1–17 (C), 5-FC-resistant strains; Lane M, a 1 kb DNA ladder plus (0.1–10.0 kb), or a 100-bp molecular weight marker (0.1–1.5 kb). For more details regarding the estimated lengths of the PCR products amplified from the genome, please see Table S2. **Table S1.** Primer pairs used in this study. Table S2. Estimated lengths of the PCR fragments that were amplified from each strain.

## Data Availability

All data supporting the claims of this manuscript are presented and made available in this manuscript. The wild-type *P. ostreatus* strain PC9 (CECT20311) is available from Spanish Type Culture Collection. The *P. ostreatus* strain 20b (Salame et al. 2012) was a kind gift of Prof. Yitzhak Hadar and Dr. Tomer M Salame, Hebrew University of Jerusalem, Israel. The base plasmid used for experiments herein; pCcPef3-126 (Sugano et al. 2017) was a kind gift of Professor Keishi Osakabe, Tokushima University, Japan.

## Abbreviations

5-FC: 5-Fluorocytosine
5-FOA: 5-Fluoroorotic acid
*Cbx*^*R*^: Carboxin resistance
CRISPR/Cas9: Clustered regularly interspaced short palindromic repeat/CRISPR-associated protein 9
DSB: Double-strand DNA break
*fcy1*: Cytosine deaminase
HDR: Homology-directed repair
hph: Hygromycin phosphotransferase
NHEJ: Non-homologous end joining
NLS: Nuclear localization sequences
*pyrG*: Orotidine 5′-phosphate decarboxylase
sgRNA: Single guide RNA
wt: Wild-type
YMG: Yeast and malt extract with glucose
YMGUU: YMG supplemented with 0.18 mM uracil and 20 mM uridine

## References

[CR1] Bhushan A, Kulshreshtha M (2018). The medicinal mushroom *Agaricus bisporus*: review of phytopharmacology and potential role in the treatment of various diseases. J Nat Sci Biol Med.

[CR2] Chakravarty B (2011). Trends in mushroom cultivation and breeding. Aust J Agri Eng.

[CR3] Corrêa RCG, Brugnari T, Bracht A, Peralta RM, Ferreira ICFR (2016). Biotechnological, nutritional and therapeutic uses of *Pleurotus* spp. (oyster mushroom) related with its chemical composition: a review on the past decade findings. Trends Food Sci Technol.

[CR4] Cummings WJ, Celerin M, Crodian J, Brunick LK, Zolan ME (1999). Insertional mutagenesis in *Coprinus cinereus*: use of a dominant selectable marker to generate tagged, sporulation-defective mutants. Curr Genet.

[CR5] Dai Y, Su W, Yang C, Song B, Li Y, Fu Y (2017). Development of novel polymorphic EST-SSR markers in bailinggu (*Pleurotus tuoliensis*) for crossbreeding. Genes.

[CR6] Doench JG, Hartenian E, Graham DB, Tothova Z, Hegde M, Smith I, Sullender M, Ebert BL, Xavier RJ, Root DE (2014). Rational design of highly active sgRNAs for CRISPR-Cas9-mediated gene inactivation. Nat Biotechnol.

[CR8] Engler C, Kandzia R, Marillonnet S (2008). A one pot, one step, precision cloning method with high throughput capability. PLoS One.

[CR7] Engler C, Gruetzner R, Kandzia R, Marillonnet S (2009). Golden gate shuffling: a one-pot DNA shuffling method based on type IIs restriction enzymes. PLoS One.

[CR9] Gregori ASM, Pohleven J (2007). Cultivation techniques and medicinal properties of *Pleurotus* spp. Food Technol Biotechnol.

[CR10] Ishino Y, Shinagawa H, Makino K, Amemura M, Nakata A (1987). Nucleotide sequence of the *iap* gene, responsible for alkaline phosphatase isozyme conversion in *Escherichia coli*, and identification of the gene product. J Bacteriol.

[CR11] Israilides C, Kletsas D, Arapoglou D, Philippoussis A, Pratsinis H, Ebringerová A, Hríbalová V, Harding SE (2008). In vitro cytostatic and immunomodulatory properties of the medicinal mushroom *Lentinula edodes*. Phytomedicine.

[CR12] Jan Vonk P, Escobar N, Wösten HAB, Lugones LG, Ohm RA (2019). High-throughput targeted gene deletion in the model mushroom *Schizophyllum commune* using pre-assembled Cas9 ribonucleoproteins. Sci Rep.

[CR13] Jinek M, Chylinski K, Fonfara I, Hauer M, Doudna JA, Charpentier E (2012). A programmable dual-RNA-guided DNA endonuclease in adaptive bacterial immunity. Science.

[CR14] Kirk P, Cannon P, Stalpers J, Minter DW (2008). Dictionary of the fungi.

[CR15] Kües U, James TY, Heitman J, Pöggeler S, Wöstemeyer J (2011). 6 Mating type in basidiomycetes: unipolar, bipolar, and tetrapolar patterns of sexuality. Evolution of fungi and fungal-like organisms.

[CR16] Larraya LM, Pérez G, Peñas MM, Baars JJ, Mikosch TS, Pisabarro AG, Ramírez L (1999). Molecular karyotype of the white rot fungus Pleurotus ostreatus. Appl Environ Microbiol.

[CR17] Mahaffey H, Taheripour F, Tyner WE (2016). Evaluating the economic and environmental impacts of a global GMO ban. J Environ Prot.

[CR18] Muraguchi H, Ito Y, Kamada T, Yanagi SO (2003). A linkage map of the basidiomycete *Coprinus cinereus* based on random amplified polymorphic DNAs and restriction fragment length polymorphisms. Fungal Genet Biol.

[CR19] Nakazawa T, Ando Y, Kitaaki K, Nakahori K, Kamada T (2011). Efficient gene targeting in ∆Ccku70 or ∆Cclig4 mutants of the agaricomycete Coprinopsis cinerea. Fungal Genet Biol.

[CR20] Nakazawa T, Izuno A, Kodera R, Miyazaki Y, Sakamoto M, Isagi Y, Honda Y (2017). Identification of two mutations that cause defects in the ligninolytic system through an efficient forward genetics in the white-rot agaricomycete *Pleurotus ostreatus*. Environ Microbiol.

[CR21] Nakazawa T, Tsuzuki M, Irie T, Sakamoto M, Honda Y (2016). Marker recycling via 5-fluoroorotic acid and 5-fluorocytosine counter-selection in the white-rot agaricomycete *Pleurotus ostreatus*. Fungal Biol.

[CR22] Nguyen DX, Nakazawa T, Myo G, Inoue C, Sakamoto M, Honda Y (2020). A promoter assay system using gene targeting in agaricomycetes *Pleurotus ostreatus* and *Coprinopsis cinerea*. J Microbiol Methods.

[CR23] Okuda Y, Murakami S, Matsumoto T (2009). Development of STS markers suitable for marker-assisted selection of sporeless trait in oyster mushroom, *Pleurotus pulmonarius*. Breed Sci.

[CR24] Qin H, Xiao H, Zou G, Zhou Z, Zhong J-J (2017). CRISPR-Cas9 assisted gene disruption in the higher fungus *Ganoderma* species. Process Biochem.

[CR25] Rao PS, Niederpruem DJ (1969). Carbohydrate metabolism during morphogenesis of *Coprinus lagopus* (*sensu* Buller). J Bacteriol.

[CR26] Raudaskoski M, Kothe E (2010). Basidiomycete mating type genes and pheromone signaling. Eukaryot Cell.

[CR27] Salame TM, Knop D, Levinson D, Yarden O, Hadar Y (2013). Redundancy among manganese peroxidases in *Pleurotus ostreatus*. Appl Environ Microbiol.

[CR28] Salame TM, Knop D, Tal D, Levinson D, Yarden O, Hadar Y (2012). Predominance of a versatile-peroxidase-encoding gene, *mnp4*, as demonstrated by gene replacement via a gene targeting system for *Pleurotus ostreatus*. Appl Environ Microbiol.

[CR30] Song R, Zhai Q, Sun L, Huang E, Zhang Y, Zhu Y, Guo Q, Tian Y, Zhao B, Lu H (2019). CRISPR/Cas9 genome editing technology in filamentous fungi: progress and perspective. Appl Microbiol Biotechnol.

[CR31] Sonnenberg A, Baars JJP, Kerrigan RW (2008) Mushroom breeding: hurdles and challenges. In: Paper presented at the 6th international conference on mushroom biology and mushroom products, Bonn, Germany, 29 September–3 October 2008

[CR32] Sugano SS, Suzuki H, Shimokita E, Chiba H, Noji S, Osakabe Y, Osakabe K (2017). Genome editing in the mushroom-forming basidiomycete *Coprinopsis cinerea*, optimized by a high-throughput transformation system. Sci Rep.

[CR33] Waltz E (2016). Gene-edited CRISPR mushroom escapes US regulation. Nature.

[CR34] Wang Q, Coleman JJ (2019). CRISPR/Cas9-mediated endogenous gene tagging in *Fusarium oxysporum*. Fungal Genet Biol.

[CR35] Wang P-A, Xiao H, Zhong J-J (2020). CRISPR-Cas9 assisted functional gene editing in the mushroom *Ganoderma lucidum*. Appl Microbiol Biotechnol.

[CR36] Xiao A, Cheng Z, Kong L, Zhu Z, Lin S, Gao G, Zhang B (2014). CasOT: a genome-wide Cas9/gRNA off-target searching tool. Bioinformatics.

[CR40] Yamasaki F, Nakazawa T, Sakamoto M, Honda Y (2021). Molecular breeding of sporeless strains of Pleurotus ostreatus using a non-homologous DNA end-joining defective strain. Mycol Prog.

[CR37] Yoav S, Salame TM, Feldman D, Levinson D, Ioelovich M, Morag E, Yarden O, Bayer EA, Hadar Y (2018). Effects of *cre1* modification in the white-rot fungus *Pleurotus ostreatus* PC9: altering substrate preference during biological pretreatment. Biotechnol Biofuels.

[CR38] Zhang YJ, Zhang S, Liu XZ, Wen HA, Wang M (2010). A simple method of genomic DNA extraction suitable for analysis of bulk fungal strains. Lett Appl Microbiol.

[CR39] Zolan ME, Pukkila PJ (1986). Inheritance of DNA methylation in *Coprinus cinereus*. Mol Cell Biol.

