# Supplementary material for: Efficient genome editing with CRISPR/Cas9 in Pleurotus ostreatus
Source: AMB Express. 2021 Feb 20;11:30. doi: 10.1186/s13568-021-01193-w (PMC7897337; doi:10.1186/s13568-021-01193-w)
Supplement: Supplementary file 1 — Additional file 1: Fig. S1. A plasmid map of the pCcPef3-126 plasmid shows the components of the construct. Fig. S2. Agarose gel electrophoresis. Genomic PCR experiments examining/verifying fcy1 (A) or pyrG (B) mutation. (C) Genomic PCR experiments confirming gene replacement in the 5-FC-resistant strains obtained after introducing the pCcPef3-126-fcy1sg2 plasmid with the donor DNA templates. Lane WT, the parental strain PC9 (A and B) and 20b (C) as a positive control; Lanes 1–10 (A and B), 5-FC- and 5-FOA- resistant strains, respectively; Lanes 1–17 (C), 5-FC-resistant strains; Lane M, a 1 kb DNA ladder plus (0.1–10.0 kb), or a 100-bp molecular weight marker (0.1–1.5 kb). For more details regarding the estimated lengths of the PCR products amplified from the genome, please see Table S2. Table S1. Primer pairs used in this study. Table S2. Estimated lengths of the PCR fragments that were amplified from each strain. [file 13568_2021_1193_MOESM1_ESM.docx]

AMB Express

**Efficient genome editing with CRISPR/Cas9 in *Pleurotus ostreatus***

Tatpong Boontawon^1^, Takehito Nakazawa^1^, Chikako Inoue^1^, Keishi Osakabe^2^, Moriyuki Kawauchi^1^, Masahiro Sakamoto^1^, Yoichi Honda^1^*

^1^Graduate School of Agriculture, Kyoto University, Kyoto 606-8502, Japan

^2^Graduate School of Technology, Industrial and Social Sciences, Tokushima University,

Tokushima 770-8503, Japan

Corresponding author: Yoichi Honda

Tel: +81 75 753 6463

Fax: +81 75 753 6471

E-mail: honda.yoichi.5n@kyoto-u.ac.jp

**Supplementary data**

**
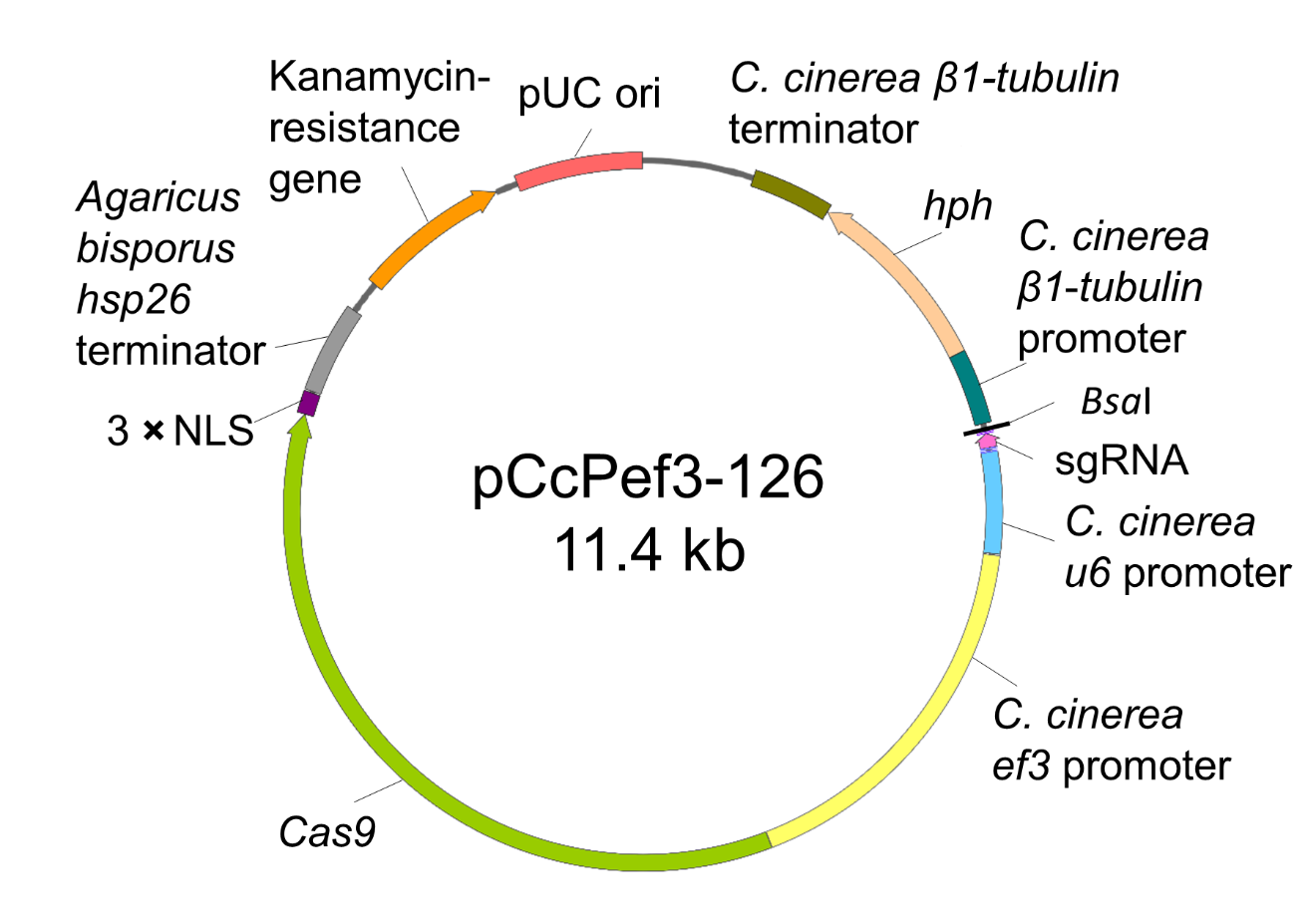
**

**Fig. S1.** A plasmid map of the pCcPef3-126 plasmid shows the components of the construct.

**
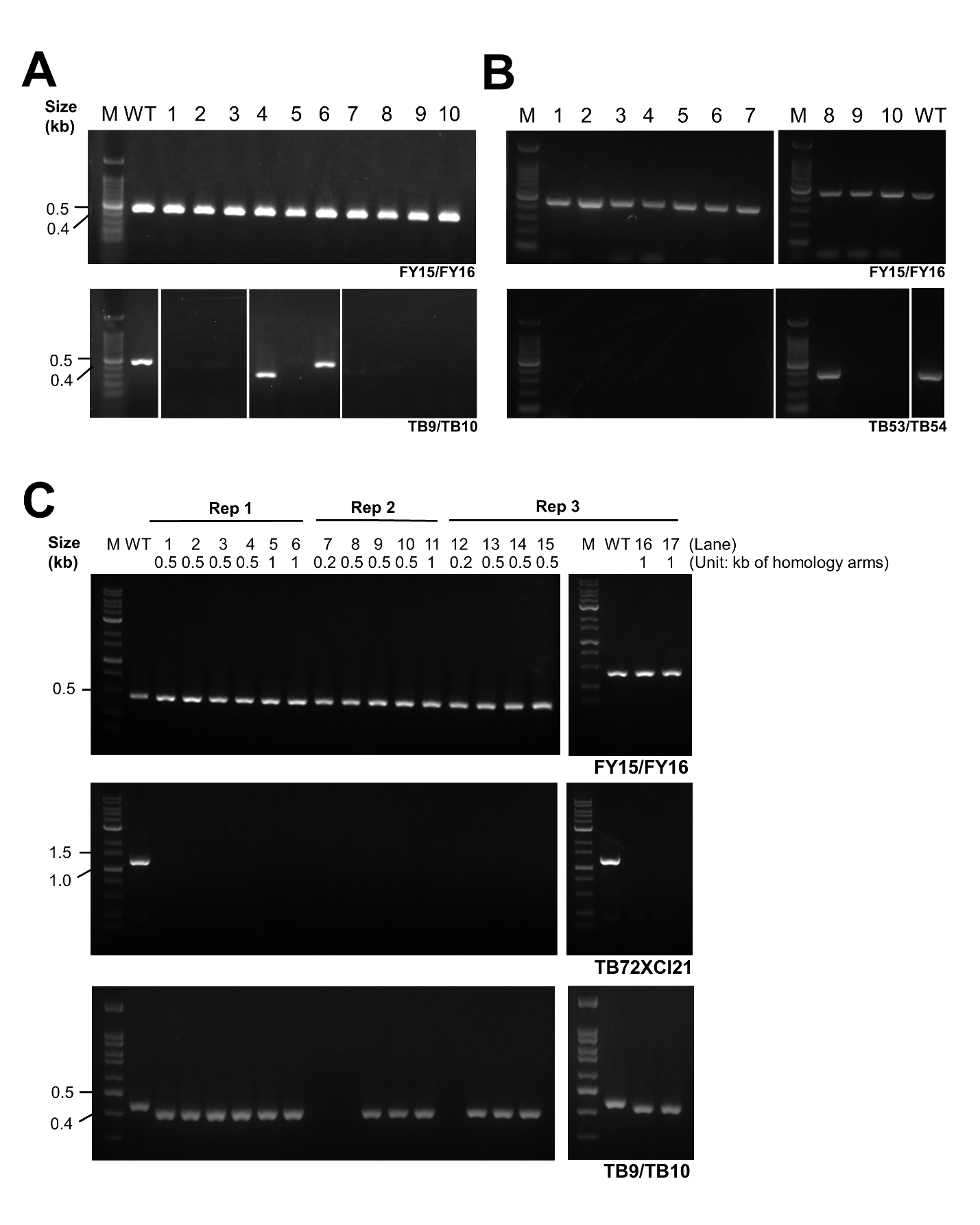
**

**Fig. S2.** Agarose gel electrophoresis. Genomic PCR experiments examining/verifying *fcy1* (A) or *pyrG* (B) mutation. (C) Genomic PCR experiments confirming gene replacement in the 5-FC-resistant strains obtained after introducing the pCcPef3-126-*fcy1*sg2 plasmid with the donor DNA templates. Lane WT, the parental strain PC9 (A and B) and 20b (C) as a positive control; Lanes 1–10 (A and B), 5-FC- and 5-FOA- resistant strains, respectively; Lanes 1–17 (C), 5-FC-resistant strains; Lane M, a 1 kb DNA ladder plus (0.1–10.0 kb), or a 100-bp molecular weight marker (0.1–1.5 kb). For more details regarding the estimated lengths of the PCR products amplified from the genome, please see Table S2.

**Table S1.** Primer pairs used in this study

| Primer | Sequence (5' to 3') |
| --- | --- |
| CI19 | GATTGTTGCGCCTCTTGAAACGCGA |
| CI20 | AAACTCGCGTTTCAAGAGGCGCAAC |
| CI21 | GATTGACCATCCCGAACCCGAACCA |
| CI22 | AAACTGGTTCGGGTTCGGGATGGTC |
| CI50 | TGGCGACCTGATGGTACGATG |
| CI51 | AGCGCCGATAGGGATTCCTC |
| CI52 | ATCCCTATCGGCGCTTGGCGGATGTCCTAGCCTCG |
| CI53 | TGTTCGTGACGACCTCGAGG |
| FY15 | AACCCCAAGTTCTATGCGTTG |
| FY16 | ACTTACTCTTCTGCAGCCGAC |
| TB9 | CATTGCTTTGGCGGTTCAAACG |
| TB10 | GCGTACCTCAGCGTCGTGTAC |
| TB11 | GCAGCCCTTTTCTTTCTCTTCCAGATC |
| TB12 | CGATGTCTTCATACCATTCCTACACCG |
| TB41 | GATTGTCTTCAGTAGGCCTACCCCT |
| TB42 | AAACAGGGGTAGGCCTACTGAAGAC |
| TB43 | GATTGCTTGGCAGAAATGAGCACCG |
| TB44 | AAACCGGTGCTCATTTCTGCCAAGC |
| TB53 | CGCCGCTTGTAGGAAACACAG |
| TB54 | CTGCTGCCCCATACCATCTCC |
| TB72 | CGTCACCAGTCATCGTGACC |
| TN40 | ACCCTTTCCCCCAAAATTTGGAAGC |
| TN46 | AAACGGCTTCACGGGCAGCC |

**Table S2.** Estimated lengths of the PCR fragments that were amplified from each strain.

| Primer set | Strain/ Function | Estimated length (bp) |
| --- | --- | --- |
| CI19/CI20 | *fcy1*sg1 | - |
| CI21/CI22 | *fcy1*sg2 | - |
| TB41/TB42 | *pyrG*sg1 | - |
| TB43/TB44 | *pyrG*sg2 | - |
| FY15/FY16 | Wild-type^1^ / positive control | 430 |
|  | Mutant / positive control | 430 |
| TB9/TB10 | Wild-type^1^ | 432 |
|  | *fcy1* mutant via NHEJ | Small indels of nucleotide, or not amplified |
|  | *fcy1* mutant via HDR | 407 |
| TB53/TB54 | Wild-type^1^ | 356 |
|  | *pyrG* mutant | Small indels of nucleotide, or not amplified |
| TB9/TN40 | Wild-type^1^ | Not amplified |
|  | *fcy1* mutant | unpredictable, or not amplified |
| TB9/TN46 | Wild-type^1^ | Not amplified |
|  | *fcy1* mutant | unpredictable, or not amplified |
| TB10/TN40 | Wild-type^1^ | Not amplified |
|  | *fcy1* mutant | unpredictable, or not amplified |
| TB10/TN46 | Wild-type^1^ | Not amplified |
|  | *fcy1* mutant | unpredictable, or not amplified |
| TB53/TN40 | Wild-type^1^ | Not amplified |
|  | *pyrG* mutant | unpredictable, or not amplified |
| TB53/TN46 | Wild-type^1^ | Not amplified |
|  | *pyrG* mutant | unpredictable, or not amplified |
| TB54/TN40 | Wild-type^1^ | Not amplified |
|  | *pyrG* mutant | unpredictable, or not amplified |
| TB54/TN46 | Wild-type^1^ | Not amplified |
|  | *pyrG* mutant | unpredictable, or not amplified |
| CI50/CI51 | 5’-upstream of *fcy1* | 1038 |
| CI52/CI53 | 3’-downstream of *fcy1* | 1048 |
| CI50/CI53 | donor DNA with homology arms of 1 kb | 2071 |
| TB11/TB12 | donor DNA with homology arms of 0.5 kb | 1008 |
| TB9/TB10 | donor DNA with homology arms of 0.2 kb | 407 |
| TB72/CI21 | Wild-type^1^ | 1226 |
|  | *fcy1* mutant via HDR | Not amplified |

^1^PC9 and 20b host strains
